# Mechanistic insights into pyrolysis temperature-dependent lead (Pb) stabilization in phytoremediation residue-derived biochar

**DOI:** 10.3389/fchem.2025.1705662

**Published:** 2025-11-12

**Authors:** Jin Liu, Yangyang Wang, Jun Pang, Jingao Wang, Tongtong Li, Lei Wang

**Affiliations:** 1 School of Materials and Environmental Engineering, Shenzhen Polytechnic University, Shenzhen, China; 2 Shenzhen Minghe Science and Technology Company Limited, Shenzhen, China; 3 College of Art, Shandong Agricultural University, Tai’an, China; 4 Technical Centre for Soil, Agriculture and Rural Ecology and Environment, Ministry of Ecology and Environment, Beijing, China

**Keywords:** phytoremediation residue, lead (Pb), pyrolysistemperature, stabilization, ecological risk

## Abstract

The substantial generation of hazardous, metal-enriched biomass residues poses significant risks of secondary contamination, presenting a critical bottleneck to the broader implementation of phytoremediation that urgently requires effective treatment solutions. This study addressed this challenge by pyrolyzing Pb-enriched biomass (BM_Pb_) across a temperature range (300 °C–700 °C) to produce Pb-enriched biochar (BC_Pb_), evaluating its efficacy for safe residue management. The results demonstrated that pyrolysis effectively reduced the volume of BM_Pb_, and the produced BC_Pb_ significantly enriched and immobilized Pb. Element analysis revealed distinct stabilization mechanisms: Pb_2_(P_4_O_12_) and PbCO_3_ precipitation dominated Pb immobilization at 400 °C, whereas Pb_3_(CO_3_)_2_(OH)_2_, Pb_2_(P_4_O_12_), and NaAlSiO_4_ became predominant at temperatures ≥500 °C. Sequential extraction of Pb (BCR) demonstrated a consistent decline in the more labile Pb fractions (exchangeable, F1, and reducible, F2) with increasing pyrolysis temperature, concurrent with a significant increasing in the stable fractions (oxidizable, F3, and residual, F4). Notably, the combined F1+F2 fraction decreased substantially (17% at 700 °C), while the stable F3+F4 fraction increased correspondingly (83% at 700 °C), indicating markedly reduced Pb bioavailability and ecological risk at elevated temperatures. Leaching tests confirmed that Pb release from all BC_Pb_ samples remained well below relevant regulatory thresholds when the pH higher than 2 (<9.98 mg·g^-1^ vs. 10.0 mg·g^-1^), with leaching susceptibility inversely related to pyrolysis temperature. Soil simulation experiments further indicated a conversion of bioavailable Pb (F1+F2) in BC_Pb_-amended systems towards stable forms (F3+F4), confirming low ecological risk. Overall, these findings suggested that pyrolysis of BM_Pb_ at temperatures above 500 °C shows great promise as an effective and safe method for treating phytoremediation residues, demonstrating high stability and low ecological risk to both water and soil environments under most natural conditions, though careful management is required under extreme acidic scenarios.

## 1 Introduction

Lead (Pb) is a major heavy metal pollutant in soil, widely produced in smelting, chemical and electroplating industries. Due to the significant bioaccumulation and biomagnification, even trace concentrations of Pb exposured in soil posed serious threats to animal and human health through trophic chains, resulting in diseases such as anemia and various neurological symptoms. As an inorganic pollutant, Pb is difficult to biodegrade. Therefore, phytoremediation is considered as an effective technology for removing Pb from soils due to the advantages of ecological friendliness and cost effectiveness (Khan et al., 2016; Wang and Delavar, 2023). Consequently, large amount of Pb-enriched biomass (BM_Pb_) are generated. For example, Pb content in Brassica juncea biomass can reach up to 10,000.00–15000.00 mg ⋅ kg^-1^ (Pérez-Esteban et al., 2014), and the dry weight of harvested Brassica juncea biomass is up to 6 t·hm^-2^ per growth season (Blaylock et al., 1997). The inappropriate disposal of these phytoremediation residue can led to Pb leakage and secondary pollution in surrounding environments. Currently, several treatment technologies, such as composting, landfilling, ashing and incineration, have been developed for BM_Pb_ (Jin et al., 2016). While these technologies temporarily achieve the disposal of BM_Pb_, there remains a high risk of secondary Pb leakage. Thus, the proper management of harvested BM_Pb_ is a key constraint affecting the implementation of phytoremediation engineering.

Previous research has shown that biochar can be derived from BM_Pb_ through slow pyrolysis under limited oxygen or anaerobic conditions (N_2_, Ar or CO_2_ atmosphere) at 300 °C–1,000 °C (Ok et al., 2015). After pyrolysis, the volume of phytoremediation residue is reduced by more than 90%, and heavy metals are concentrated and immobilized within the biochar matrix. (Stals et al., 2010a; Stals et al., 2010b). For instance, pyrolysis of Pb-polluted Typha angustifolia biomass can effectively immobilize 98.8% of Pb in biochar, with only a small amount of Pb retained in the incidental bio-oil and non-condensable components (Liu et al., 2011). The concentration of heavy metals in biochar increased with rising pyrolysis temperature (Zielińska and Oleszczuk, 2015). Biochar is capable of immobilizing metal (loid) s through precipitation with inorganic constituents (such as carbonate, phosphate, and silicate), adsorption and complexation with oxygen-containing functional groups. Therefore, preparing biochar from BM_Pb_, labeled as Pb-enriched biochar (BC_Pb_), was a viable strategy for treating phytoremediation residues. Significantly, the pyrolysis temperature affect various physicochemical characteristics of biochar, such as the pH level, surface area, and stability (Jiang et al., 2025; Liu et al., 2025). Generally, the yield and H/O content of biochar decrease with the increasing pyrolysis temperature. In contrast, the levels of aromatization and graphitization show an increasing trend (Li et al., 2019). Initially, the pyrolysis process is governed by dehydrogenation and dehydration reactions, resulting in increasing aromatization and stability of biochar (Pariyar et al., 2020). Meanwhile, the high molecular weight components in biomass undergo depolymerization at temperatures exceeding 300 °C (Keiluweit et al., 2010). At temperatures exceeding 700 °C, the highly stable lignin macromolecules start to decompose, leading to a reduction of the O/C and H/C ratios and facilitating the unordered, graphite-like microcrystalline structure of biochar (McBeath et al., 2011). In addition, high pyrolysis temperatures enhance the alkalinity and ash content of straw biochar (Zhang X. et al., 2020). Therefore, gaining a more comprehensive insight into how pyrolysis temperature related to the characteristics of Pb in BC_Pb_ was crucial.

The presence of biochar in an oxidizing environment can lead to alterations to its oxygen-related functional groups, inorganic mineral composition, pore architecture, and other characteristics. (Ji et al., 2022; Zhang X. et al., 2020). Pb may leach from biochar during rainfall, acid rain and biogeochemical interactions, which is also affected by soil pH, organic matter and moisture (Changotra et al., 2017; Gul et al., 2015). Furthermore, the migration, bioavailability and toxicity of Pb are associate with its chemical forms in BC_Pb_, which play the most significant impact on the ecological risk of BC_Pb_ in soils (Changotra et al., 2017; Xu et al., 2024). BC_Pb_ may pose potential ecological risks during long-term storage in natural environments. Pyrolysis temperature significantly impacts the physicochemical properties of BC_Pb_ (He et al., 2023; Wang et al., 2015; Wang et al., 2018). Thus, the comprehensive evaluation of stabilization performance of BC_Pb_ and the leaching risk of Pb from BC_Pb_ is necessary before the widespread application of pyrolysis in treating BM_Pb_.

*Iris sibirica* L. is a potential hyperaccumulator for phytoremediation of Pb-polluted regions. This study provided the first comprehensive investigation into the pyrolysis temperature-dependent transformation of Pb speciation and stabilization mechanisms in biochar derived from *Iris sibirica L.* residues. We aimed to elucidate how specific pyrolysis temperatures direct Pb into distinct, stable mineral phases (e.g., phosphates, carbonates) and to quantitatively evaluated the resulting environmental stability through multi-condition leaching tests and soil incubation. In this research, the leaching behavior of BC_Pb_ was examined by subjecting BM_Pb_ extracted from *Iris sibirica* L. to pyrolysis at varying temperatures. Leaching experiments of the prepared BC_Pb_ were conducted under neutral, acidic, alkaline and oxidative conditions. Additionally, soil simulation experiments were performed. These studies aimed to analyse (I) the influence of pyrolysis temperatures on the morphological structure, chemical forms and distribution of Pb in BC_Pb_, (II) the leaching behaviors of Pb from BC_Pb_ under acidic, alkaline and oxidative conditions, and (III) the interactions between the bioavailable fractions of BC_Pb_ and soil environments. Overall, these results could enhance our comprehension of BC_Pb_’s stability and demonstrate the feasibility of addressing BM_Pb_ through pyrolysis.

## 2 Materials and methods

### 2.1 Preparation of samples

To obtain the uniform BM_Pb_, *Iris sibirica* L. was hydroponically cultivated with a week pre-incubation and Pb^2+^ solution (0, 300.00, and 500.00 mg ⋅ L^-1^) for 2 months (Supplementary Texts A1, A2). Biochar samples were obtained by pyrolysis in a tube muffle furnace under N_2_ atmosphere (flow rate of 200 mL·min^-1^) at 300, 400, 500, 600 °C and 700 °C for 2 h, with a heating rate of 10 °C·min^-1^. The biomass was washed, oven-dried at 80 °C, and ground to pass through a 100-mesh sieve before pyrolysis. More detailed information described in Supplementary Text A3. The biomass under Pb hydroponic conditions at concentrations of 0, 300.00, and 500.00 mg·L^-1^ were designated as BM_CK_, BM_Pb_, and BM_H_, respectively, with their corresponding biochars at X (300, 400, 500, 600 °C and 700 °C) pyrolysis temperature were marked as ${BC}_{CK}^{X}{,BC}_{PB}^{X}$ and ${BC}_{H}^{X}$ .

### 2.2 Characterization of BC_Pb_ samples

Scanning Electron Microscopy (SEM) system with Energy Dispersive X-ray Spectrometry (EDS) Elemental Mapping (model SU8000, Hitachi High-Technologies Corporation, Japan) was utilized to analyze the micro-topography and elemental composition of as prepared samples. Fourier Transform Infrared Spectroscopy (FT-IR, model NICOLET 5700, Thermo Fisher Scientific, USA) was employed to identify the functional groups of biochar within the wavelength range of 400.00–4,000.00 cm^-1^ at a resolution of 1.00 cm^-1^. The crystalline structure of the biochar was characterized using an X-ray diffractometer (XRD, model D/Max 2,500, Rigaku Corporation, Japan).

### 2.3 Pb total content and speciation

To assess the Pb content, biochar and soil samples were analyzed by Inductively Coupled Plasma Mass Spectrometer (ICP-MS) (Agilent 7500cx), using 2% HNO_3_ solution as the blank reference (Supplementary Text A4). Certified reference materials (GBW07407) and spike recovery tests were performed for quality assurance/quality control (QA/QC).

Four Pb species in biochar and soil samples were measured by BCR sequential extraction method, including F1 fraction (exchangeable), F2 fraction (reducible), F3 fraction (oxidizable), and F4 fraction (residual). The accessibility of heavy metals for biological uptake and ecological risks of these fractions follow the order: exchangeable > reducible > oxidizable > residual. The specific extraction steps of the BCR sequential extraction method were described in Supplementary Text A5. The recovery rates of the BCR sequential extraction, calculated as (F1+F2+F3+F4)/Total Pb, were between 90% and 102% for all samples, which is within the acceptable criteria for this method.

### 2.4 Simulated leaching of BC_Pb_ and soil simulated experiment

The leaching behaviors of Pb from BC_Pb_ in deionized water, Toxicity Characteristic Leaching Procedure (TCLP), acid, alkaline, and oxidative solutions were determined as described previously. Specific steps for the leaching experiment are detailed in Supplementary Text A6 in the Supplementary Material (SI). For the pH-dependent leaching tests, the final pH of the supernatant after the 8-h incubation was measured and recorded, as the alkalinity of biochar can shift the solution pH. Top soil samples (from 0 to 20 cm depth) were gathered located in Shunyi District, Beijing, China. Following a week-long acclimatization period in an artificial climate incubator with 28.0 °C ± 0.5 °C temperature and 16/8 h (light/dark) photoperiod conditions, 1% (w/w) of BM_CK_, BM_Pb_ and BC_Pb_ were incorporated. and cultured for 45 days. The soil was sprayed with deionized water every 2 days to maintain soil humidity (60%) during the experiments. The experiment was conducted with three replications (Supplementary Text A7).

### 2.5 Evaluation of possible ecological hazards associated with Pb in BC_Pb_

The potential ecological risk assessment index (RI), risk assessment code (RAC) and Muller geological accumulation index (I_geo_) were employed to evaluate the potential ecological risks associated with heavy metals, as calculated using the formulas provided in Supplementary Text A8. All experiments were performed in triplicate. Data were presented as mean ± standard deviation. Statistical significance was determined by one-way analysis of variance (ANOVA) followed by Tukey’s *post hoc* test using SPSS software (version 22.0). Differences were considered significant at p < 0.05.

## 3 Results and discussion

### 3.1 Ash content, yield rates and pH of BC_Pb_

The pH, ash content, and yield rates in biochar produced at various temperatures were presented in Supplementary Table SA1, Supplementary Figures SA1, SA2. Evidently, the Pb contents per biomass unit in roots were higher than those in shoot of phytoremediation residues, thus, the shoot was selected as a suitable sample in this study. The contents of Pb in BC_CK_, BC_Pb_ and BC_H_ were higher than those in corresponding biomass, and the ash content in BC_Pb_ (or BC_H_) was considerably greater than that in BC_CK_ at each pyrolysis temperature. The higher ash contents in BC_Pb_/BC_H_ were probably attributed to the thermal stability of the Pb element in phytoremediation residues (Zhang Y. et al., 2020). In BC_CK_ and BC_Pb_ obtained from 300 °C to 700 °C, the ash content rose from 14.53% to 44.27% and from 26.93% to 54.72%, whereas the yield rates obviously decreased from 47.98% to 39.4% and from 63.4% to 37.39%, respectively. Notably, at 600 °C and 700 °C, BC_Pb_ showed a lower biochar yield rate than BC_CK_, which suggested that Pb might catalyze the decomposition of biomass, thereby increasing the production of volatiles (Liu et al., 2012) (Supplementary Table SA1).

As reported, the increase of pH was primarily attributed to the elevated ash content in biochar (Perin et al., 1985). In this study, a significant positive correlation was observed (*R* ^2^ = 0.893, P = 0.019) between ash contents and pH value according to correlation analysis (Supplementary Table SA2). In detail, the pH values of BM_CK_ and BM_Pb_ were approximately neutral. After pyrolysis, the pH value of biochar consistently increased with the increasing pyrolysis temperature and reached the maximum at 700 °C, pH = 9.84 and 10.49 for BC_CK_ and BC_Pb_, respectively (Supplementary Table SA1). This result was consistent with Qian et al. (Qian et al., 2019), revealing that high temperatures could effectively increase the alkalinity of biochar, which might be beneficial to the stabilization of Pb in biochar. In addition, at the same pyrolysis temperature, BC_Pb_ always showed higher pH than BC_CK_, indicating that the introduction of Pb could increase the pH of biochar. As Yuan et al. and Zheng et al. revealed the leached alkali metals (such as Na^+^, K^+^, Mg^2+^ and Ca^2+^) would be an important factor in increasing alkalinity during the pyrolytic conversion process. In summary, as the pyrolysis temperature rose, the yield rate of BC_Pb_ significantly depressed, while the ash content and pH value tended to increase.

### 3.2 Characterization

#### 3.2.1 FTIR and XRD characterization

**FIGURE 1:**
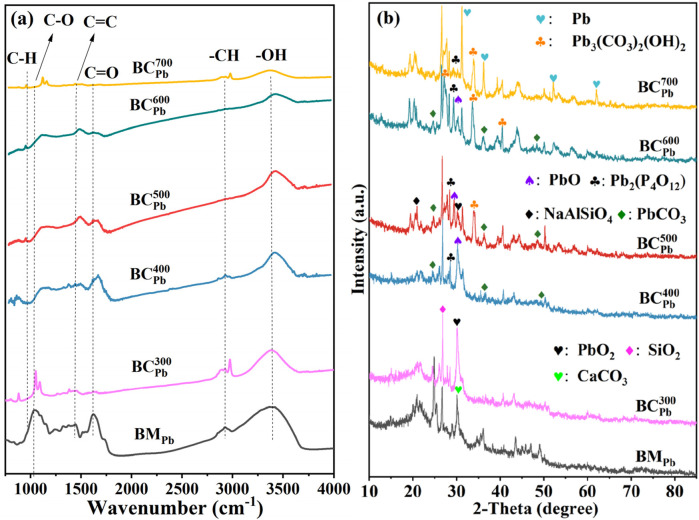
**(a)** FTIR and **(b)** XRD spectra of BM_Pb_ and ${BC}_{Pb}^{X}$ (X = 300, 400, 500, 600, 700 °C).

FTIR spectra (Figure 1a; Supplementary Figure SA3) were performed to explore the functional groups of BC_Pb_ and BC_H_. Generally, the pyrolysis temperature played a key role in influencing the functional groups present on biochar (Hossain et al., 2011). The evident diffraction peaks were detected at 881, 1,000–1,100, 1,439, 1,600, 2,916, and 3,440 cm^-1^, representing the C-H, C-O, C=C, C=O, -CH, and -OH groups, respectively. Nevertheless, a high pyrolysis temperature (exceeding 500 °C) led to the removal of functional groups from both BC_Pb_ and BC_H_. In detail, C-O bending belonging to vibration of carbonate decreased with the increasing temperature and disappeared at 500 °C (Chen et al., 2015). From 300 °C to 700 °C, the C=O stretching associated with deoxygenation (Cao et al., 2021) and the C=C stretching of conjugated olefins progressively diminished (Fan et al., 2021). Similarly, the intensity of the -OH (Afzal et al., 2018; Xia et al., 2019) and the -CH (Fan et al., 2022) decreased due to dehydration and dehydrogenation reactions. On the contrary, the intensity of C-H (Zong et al., 2020) increased wiht the increasing temperature, which was associated with the vibration of aromatic rings. A higher pyrolysis temperature facilitated the deoxygenation and dehydrogenation of aliphatic compounds, thereby promoting the formation of aromatic ring structures. These findings was consistent with the results reported by Fan et al. (Fan et al., 2022). Furthermore, the annihilation effect of Pb in biochar on the acidic surface functional groups might explain the higher pH of BC_Pb_ than BC_CK_ in this study.

The XRD analysis demonstrated that calcite (CaCO_3_) was the major peak in BM_Pb_, whereas the main peaks at 2θ = 30.0°, which corresponded to PbO_2_ in ${BC}_{\text{Pb}}^{300}$ (PDF # 50-1,430) (Zhang and Lin, 2011) (Wang et al., 2025) (Figure 1b; Supplementary Figure SA4). The intensity of PbO_2_ gradually diminished with the increasing temperature, probably due to the deoxidization ability by carbon species under anaerobic condition. Once the temperature raised to 400 °C, the peaks of PbO (PDF # 38-1,477) (Wu et al., 2022) and Pb_2_(P_4_O_12_) (PDF # 40-0208) (Rodríguez-Sánchez et al., 2017) at 2θ = 29.2° and 28.2° emerged, suggesting that the PbO_2_ in ${BC}_{Pb}^{300}$ as a precursor was reducted to PbO and transformed to phosphate precipitation in ${BC}_{Pb}^{400}$ . As reported, a substantial quantity of bioavailable phosphorus (P) was released under the alkaline and high temperature environment (Xu et al., 2020). Thereby, insoluble phosphate precipitation might be formed with PO^3-^_4_and Pb^2+^ at the biochar surface. Simultaneously, the main peaks at 2θ = 24.7°, 36.1° and 48.9° were indexed to the PbCO_3_ (PDF # 47-1734) (Fiorito et al., 2022). The three peaks about PbO, Pb_2_(P_4_O_12_) and PbCO_3_ increased with rising temperature and reach maximum intensities at 600 °C. These findings indicated that the primary forms of Pb immobilization in BC_Pb_ at temperatures above 400 °C could be attributed to phosphate and carbonate precipitation (Chang et al., 2019). In ${BC}_{\text{Pb}}^{500}$ , relatively strong diffraction peaks at 2θ = 33.9° and 20.8° were observed, matching well with Pb_3_(CO_3_)_2_(OH)_2_ (PDF # 28-0529) (Zhang et al., 2013) and NaAlSiO_4_ (PDF # 11-0220) (Chen et al., 2016). By calculation, the formation of Pb_3_(CO_3_)_2_(OH)_2_ was significantly positively correlated with the pH value (p = 0.021). At high pyrolysis temperature (≥500 °C), the alkaline property of biochar might facilitate the formation of Pb_3_(CO_3_)_2_(OH)_2_, which was accompanied by the CO_3_^2-^ and OH^−^ precipitated on the surface of BC_Pb_ (Hong et al., 2015). Similar observations were observed in BC_H_ (Supplementary Figure SA4). In addition, the high alkalinity might induce the decomposition and recombination of Si-O and Al-O, leading to the formation of NaAlSiO_4_ (Li et al., 2018). The formed NaAlSiO_4_ was benefitial to the assembling of Pb inside (Chen et al., 2014), thus reducing the mobility of Pb. Assi et al. (Assi et al., 2020) and Chang et al. (Chang et al., 2020) also reported comparable findings. Under pyrolysis temperature of 700 °C, prominent diffraction peaks were detected at angles of 31.1°, 36.2°, 52.0°, and 62.0°, corresponding to Pb^0^ (PDF # 04-0686) (Dong et al., 2020). Apart from Pb^0^, peaks of Pb_3_(CO_3_)_2_(OH)_2_, Pb_2_(P_4_O_12_) and NaAlSiO_4_ still existed, whereas the peak of PbCO_3_ disappeared due to the low thermal stability. A semi-quantitative analysis based on XRD peak areas (Supplementary Text A9; Supplementary Table SA3) further confirmed this transformation pathway, showing the progressive decrease of PbO_2_ and the rise of stable Pb(II) phosphates and carbonates, followed by the formation of Pb^0^ at 700 °C.

#### 3.2.2 SEM and EDS

**FIGURE 2:**
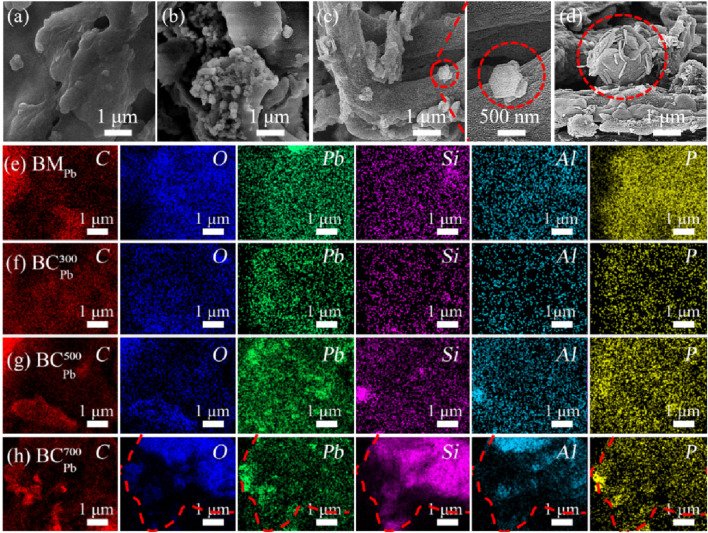
**(a–d)** SEM and **(e–h)** EDS elemental mapping images of BM_Pb_, ${BC}_{\text{Pb}}^{300}$ , ${BC}_{\text{Pb}}^{500}$ and ${BC}_{\text{Pb}}^{700}$ .

The SEM of BM_Pb_, ${BC}_{\text{Pb}}^{300}$ , ${BC}_{\text{Pb}}^{500}$ and ${BC}_{\text{Pb}}^{700}$ were shown in Figure 2. The surface of BM_Pb_ was relatively smooth, while the BC_Pb_ showed rough surfaces with highly porous structure. During pyrolysis process, organic matters were decomposed and gaseous products were released, which would form abundant pores on the surface of biochar. The pore structure could enhance the adsorption ability of biochar, thereby decreasing the mobility and biological availability of Pb in BC_Pb_ (Puga et al., 2015). Especially, single hexagonal flake-like crystals and petaloid agglomerates were respectively observed at ${BC}_{\text{Pb}}^{500}$ and ${BC}_{\text{Pb}}^{700}$ , this special crystal was considered as Pb_3_(CO_3_)_2_(OH)_2_ in previous study (Figures 2b–d). This result consistent with the XRD spectra in Figure 1b. The EDS analysis identified several elements on the biochar, including Pb, P, Si, and Al. Pb and P elements exhibited more matching signals in BC_Pb_, especially in ${BC}_{\text{Pb}}^{700}$ . These results suggested the formation of phosphate precipitates, probably contributing to the reduction of Pb mobility in biochar (Xu et al., 2018). Ahmad et al. reported that the complex of metal-phosphate precipitation was the critical reason for the reduced mobility of heavy metals (Ahmad et al., 2016). EDS results also showed obvious Si and Al signals in biochar obtained at temperature exceeding 500 °C. In line with the shape of P and Pb signals, the O, Si, and Al elements also exhibited perfect overlapping skull-like configuration in ${BC}_{\text{Pb}}^{700}$ (Figure 2h inset red lines). It could be speculated that the Pb_2_(P_4_O_12_) was mixed with the NaAlSiO_4_ under the high temperature molten state, and the stabilization effect of NaAlSiO_4_ might further promote the immobilization of Pb in BC_Pb_. This result confirmed the presence of NaAlSiO_4_ in XRD analysis. Therefore, NaAlSiO_4_ was crucial in the immobilization of Pb, which might be another potential reason contributing to the stability of Pb in biochar.

### 3.3 Influence of temperature on the content and distribution of Pb in BC_Pb_

#### 3.3.1 Total content of Pb

**FIGURE 3:**
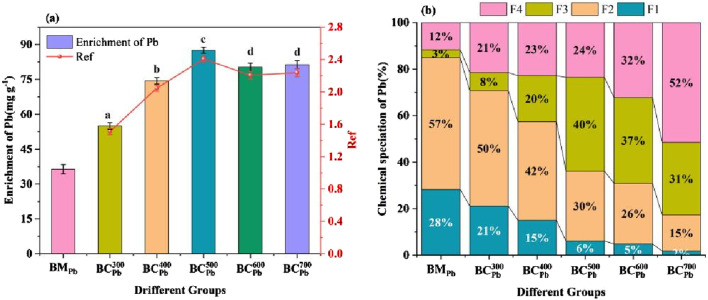
**(a)** Total concentration, **(b)** chemical speciation of Pb in BM_Pb_ and ${BC}_{\text{Pb}}^{\text{CX}}$ (X = 300, 400, 500, 600, 700 °C). For Figure **(a)**, error bars represent standard deviation (n = 3). Different lowercase letters above bars (or data points) indicate significant differences (p < 0.05) among groups.

To assess the enrichment of Pb in biochar, the total Pb concentration and Ref were identified using the microwave digestion-ICP approach (Figure 3a). The total Pb concentration in BM_Pb_, ${BC}_{\text{Pb}}^{300}$ , ${BC}_{\text{Pb}}^{400}$ , ${BC}_{\text{Pb}}^{500}$ , ${BC}_{\text{Pb}}^{600}$ and ${BC}_{\text{Pb}}^{700}$ were 36.38, 55.00, 74.43, 87.57, 80.26 and 81.40 mg ⋅ g^-1^, respectively (Figure 3a). It was evident that Pb in BM_Pb_ were constantly concentrated in BC_Pb_ with rising temperature. All the Ref values were greater than 1, demonstrating that the enrichment of Pb in BC_Pb_ were effectively improved through the pyrolysis approach. Comparable outcomes were demonstrated in BC_H_ (Supplementary Figure SA5).

#### 3.3.2 Chemical speciation of Pb

The availability and toxicity of heavy metals are primarily influenced by their specific fractions, rather than the total concentration of heavy metals in biochar. The chemical speciation distribution of Pb in BC_Pb_ was measured using the BCR extraction method (Figure 3b). The main species of Pb in BM_Pb_ were the exchangeable fraction (F1 fraction, 28%) and the reducible fraction (F2 fraction, 57%), which directly determined the ecological risks of BM_Pb_ (Chen et al., 2008). When the temperature increased from 300 °C to 700 °C, the percentage of F1 and F2 fractions of Pb dropped sharply from 21.56% to 2.12%, and from 50.42% to 15.06%, respectively. Meanwhile, the percentages of oxidizable fraction (F3 fraction) and residual fraction (F4 fraction) in BC_Pb_ gradually increased with rising temperature. Especially in ${BC}_{\text{Pb}}^{700}$ , the F3 fraction (31.13%) and F4 fraction (52.10%) residual fraction was 10.33 and 4.33 times higher than those in BM_Pb_, becoming the major fraction of Pb. These results indicated that the pyrolysis process successfully converted the bioavailable portions (F1+F2 fraction) of Pb to stable portions (F3+F4 fraction). Similar conversion were obtained in the BM_H_ and BC_H_ (Supplementary Figure SA6), revealing that increased temperature led to the immobilization of Pb in biochar, which was largely influenced by pyrolysis temperature. Although the BCR extraction method was operationally defined, the observed shift of Pb into the stable F3 and F4 fractions with increasing pyrolysis temperature was corroborated by an independent X-ray Photoelectron Spectroscopy (XPS) analysis. XPS performed on the post-BCR residue of ${BC}_{\text{Pb}}^{500}$ confirmed that the lead in these stable fractions was predominantly in the Pb^2+^ state (Supplementary Figure SA7), which was characteristic of insoluble precipitates such as phosphates and carbonates, thereby supporting the stabilization mechanism suggested by the BCR results. This result was consistent with the previous finding of Cu in biochar obtained from sludge (Jin et al., 2016).

### 3.4 Effect of temperature on the stability of Pb in BC_Pb_

#### 3.4.1 Leaching properties of Pb from BCPb in simulated solutions

**FIGURE 4:**
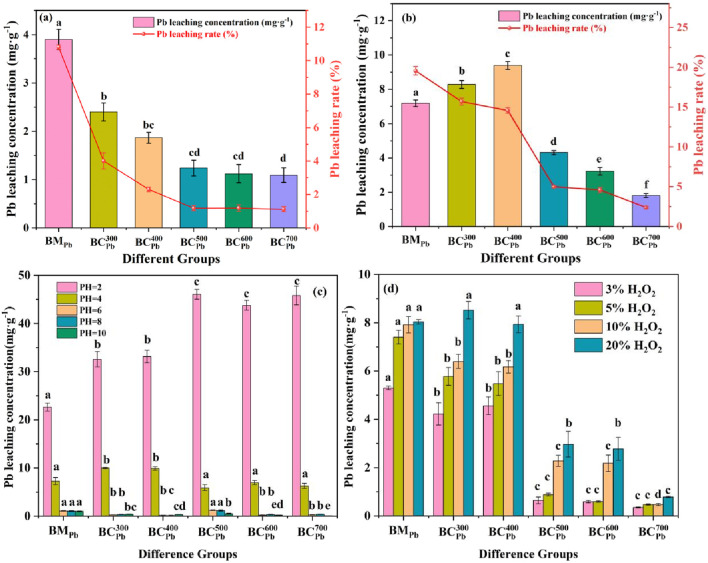
Pb leaching concentration in **(a)** deionized water experiments, **(b)** TCLP experiments **(c)** pH experiments and **(d)** H_2_O_2_ experiments of BM_Pb_ and ${BC}_{\text{Pb}}^{\text{CX}}$ (X = 300, 400, 500, 600, 700 °C). Error bars represent standard deviation (n = 3). Different lowercase letters above bars (or data points) indicate significant differences (p < 0.05) among groups. For Figure **(c,d)** the statistical comparisons and letter designations were independent for each pH and H_2_O_2_ condition.

Leaching experiments were conducted using deionized water, TCLP, pH and H_2_O_2_ condition (Figure 4; Supplementary Figure SA8), which aimed to simulate surface water or groundwater, landfill, acid rain and long-term aging circumstances (Zhang Y. et al., 2020). In the deionized water experiments, the Pb leaching concentration from BM_Pb_ was 3.90 mg·g^-1^ (Figure 4a). Comparatively, the Pb leached from ${BC}_{\text{Pb}}^{300}$ and ${BC}_{\text{Pb}}^{700}$ were 2.40 and 1.02 mg·g^-1^, respectively. With the increasing pyrolysis temperature, the leaching rate of Pb from BC_Pb_ progressively diminished, indicating that pyrolysis could effectively reduce the Pb leaching content (Zeng et al., 2018). In the TCLP experiments, the leaching concentrations and leaching rates of Pb in BC_Pb_ and BM_Pb_ were illustrated in Figure 4b. Compared with the deionized water condition, the leaching amount of Pb using the TCLP condition was approximately two folds higher (Figure 4b). This phenomenon probably due to the weak acidity of TCLP solution, which could enhance the movement and biological accessibility of Pb in BC_Pb_. The leaching amount of Pb firstly increased and then decreased from BC_Pb_ prepared under increasing pyrolysis temperatures ranging from 300 °C to 700 °C in TCLP condition. The minimal leaching rate of Pb was primarily due to the development of small, compact pores on the ${BC}_{\text{Pb}}^{500}$ surface. More stable and insoluble metal-precipitation (such as Pb_2_(P_4_O_12_), Pb_3_(CO_3_)_2_(OH)_2_ and NaAlSiO_4_ identified in XRD) inhibited the leaching of Pb as well, which was in agreement with the results reported by Jiu et al. (Jiu et al., 2015).

The pH value played a vital role in influencing the extraction of heavy metals from biochar. Therefore, the concentration of Pb leaching from BM_Pb_ and BC_Pb_ under pH = 2-10 were further evaluated. Among different pH conditions, acidic experiments (pH = 2.0 and 4.0) showed significantly higher Pb leaching concentrations from BC_Pb_ than those in weak acidic and alkaline experiments (pH = 6.0-10.0) (Figure 4c). Crucially, the final pH of the leaching solution, which was measured after the incubation (Supplementary Table SA4), provided a clear explanation for these trends. The inherent alkalinity of biochar, which increased with pyrolysis temperature, significantly buffered the acidic solutions. For instance, at an initial pH of 4.0 (simulating acid rain), the final pH in contact with ${BC}_{\text{Pb}}^{700}$ rose to approximately 7.1, effectively neutralizing the acidity and explaining the very low Pb leaching observed. Generally, at low pH (pH = 2.0-4.0), Pb ions in carbonate precipitates of BC_Pb_ could be easily dissolved (Chen et al., 2014). At high pH (pH = 6.0-10.0), Pb ions might precipitate with hydroxide anion, which then reduced the leaching amount of Pb (Mahapatra et al., 2020). Interestingly, at pH value of 2.0, the Pb leaching concentrations were exceptionally high, ranging from 43.78 to 46.06 mg·g^-1^ in ${BC}_{\text{Pb}}^{500}$ , ${BC}_{\text{Pb}}^{600}$ and ${BC}_{\text{Pb}}^{700}$ , which was probably due to the solubility characteristic of Pb^0^ under acidic conditions. At pH value of 4.0, the Pb leaching concentration at ${BC}_{\text{Pb}}^{300}$ and ${BC}_{\text{Pb}}^{400}$ were far greater than any others, which was consistent with the TCLP experiments with the same weak acidic condition. These results suggested that acid rain might increase the risk of Pb leakage from BC_Pb_. As we all known, the pH value of acid rain is about 4.2–5.6, and the Pb leaching concentrations totally conform to the pollution standard concentration (10.0 mg·g^-1^) for landfills in China (GB 16889-2008). To sum up, high leaching under strong acidic conditions (pH = 2) posed a serious environmental concern, highlighting that while BC_Pb_ was stable under most natural conditions (pH > 4), it may not be suitable for disposal in environments prone to extreme acidification, such as acid mine drainage sites, without additional safeguards. By exploring the leaching conditions of Pb in various extreme environments provided a basis for subsequently avoiding risks associated with such extreme conditions.

In the H_2_O_2_ experiments, the Pb leaching concentration from BC_Pb_ still revealed a decreasing tendency as the pyrolysis temperature rose (Figure 4D), which aligned with the tendency in deionized water leaching experiments. Especially, When the concentration of H_2_O_2_ was 5%, the concentrations of leachable Pb from ${BC}_{\text{Pb}}^{500}$ decreased by 93.77% and 94.38% compared with ${BC}_{\text{Pb}}^{300}$ and ${BC}_{\text{Pb}}^{400}$ respectively. At the 700 °C pyrolysis temperature, the concentration of Pb leaching from ${BC}_{\text{Pb}}^{700}$ was reduced to 1/10 of that of BM_Pb_ when the H_2_O_2_ concentration ranged from 3% to 10%. The findings demonstrated that the high pyrolysis treatment could significantly suppress the Pb leaching from BC_Pb_ exposed to oxidative environments (Jia et al., 2025). This might be ascribed to the stabilization of Pb_3_(CO_3_)_2_(OH)_2_ and Pb_2_(P_4_O_12_) formed at pyrolysis temperature above 500 °C, which prevented the release of Pb. In addition, for BM_Pb_ and BC_Pb_ samples, the Pb leaching concentration gradually increased with rising H_2_O_2_ concentration (Figure 4d). Overall, the ${BC}_{\text{Pb}}^{500}$ demonstrated greater economic efficiency for storage in rainfall, acid rain, and landfill environments compared to other samples, while ${BC}_{\text{Pb}}^{500}$ exhibited better performance for long-term aging under natural conditions.

#### 3.4.2 Exposure of BC_Pb_ to natural soil experiments

**FIGURE 5:**
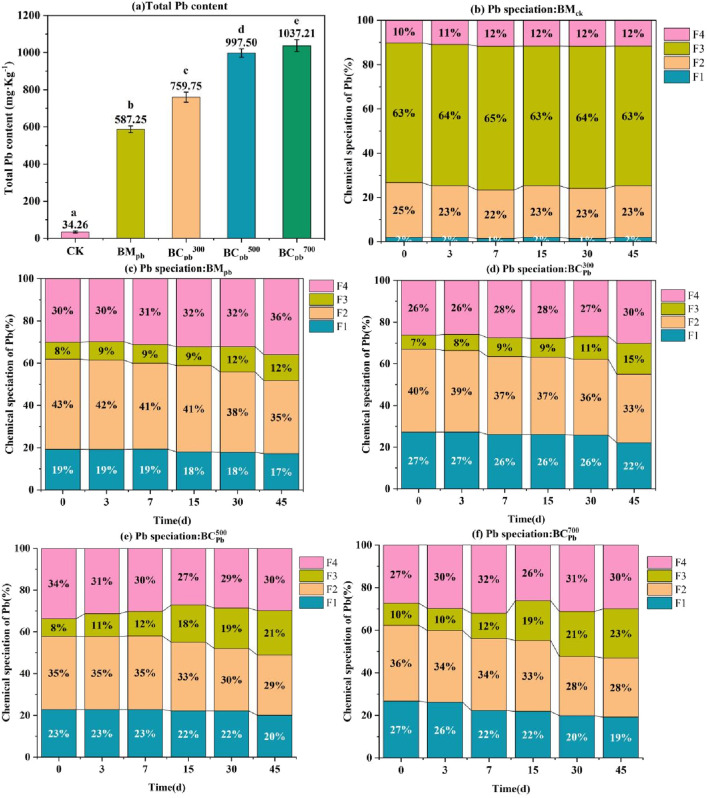
**(a)** The total Pb content in soils. **(b–f)** The proportions of different Pb species of BM_CK_, BM_Pb_, ${BC}_{\text{Pb}}^{\text{CX}}$ (X = 300, 500, 700 °C) groups during 45 days exposure. For Figure **(a)** error bars represent standard deviation (n = 3). Different lowercase letters above bars (or data points) indicate significant differences (p < 0.05) among groups.

To determine the leaching content and chemical speciation of Pb during the storage of BM_Pb_, and BC_Pb_ in natural environments, BM_CK_, BM_Pb_, ${BC}_{\text{Pb}}^{300}$ , ${BC}_{\text{Pb}}^{500}$ and ${BC}_{\text{Pb}}^{700}$ were added into natural soil and subsequently cultured for 45 days. During the cultivation process, the pH of the soil in the blank group remained unchanged (Supplementary Table SA5). The addition of BM_Pb_ or BC_Pb_ in soil caused a minor rise in pH levels, resulting in the precipitation of heavy metals such as Pb (Cao and Harris, 2010). As shown in Figure 5a, the baseline Pb content in the soil was relatively low, measuring at 34.26 mg ⋅ kg^-1^. The total Pb concentrations in the soil after exposure to BM_Pb_, ${BC}_{\text{Pb}}^{300}$ , ${BC}_{\text{Pb}}^{500}$ and ${BC}_{\text{Pb}}^{700}$ were 587.25, 759.75, 997.50 and 1,037.21 mg ⋅ kg^-1^ (Figure 5a), respectively, which were positively correlated with the Pb content in BM_Pb_ and BC_Pb_ (Figure 3a). In the control group with BM_CK_, the proportions of different Pb species remained stable during the 45 days exposure (Figure 5b). Differentially, after adding BM_Pb_ and BC_Pb_, the content of F1+F2 fraction decreased and the content of F3+F4 fraction increased along with the exposure time, suggesting the significant transformation from a bioavailable state to a stable state. Attribute to the high pH as well as the large amount of humic substances in soil, the toxicity and migration of Pb were remarkably reduced by forming insoluble complexes with organic compounds (Rodriguez-Vila et al., 2015). After exposure for 45 days, the content of F3+F4 fraction reached to 48.35%, 46.85%, 50.78% and 54.86% in groups with BM_Pb_, ${BC}_{\text{Pb}}^{300}$ , ${BC}_{\text{Pb}}^{500}$ and ${BC}_{\text{Pb}}^{700}$ , respectively (Figures 5c–f). In contrast, the bioavailable Pb state (F1+F2 fraction) dropped by 10.13%, 12.58%, 8.67%, and 15.65% in comparison to day 0, respectively. Especially, the content of F3+F4 fractions in ${BC}_{\text{Pb}}^{700}$ was larger than ${BC}_{\text{Pb}}^{300}$ and ${BC}_{\text{Pb}}^{500}$ after 45 days of incubation. In ${BC}_{\text{Pb}}^{700}$ , F4 fraction first increased and then decreased, while the content of F3 fraction increased during the 45 days. As reported, the inorganic constituents of biochar were more crucial for the immobilization of Pb, and the Pb^0^ within ${BC}_{\text{Pb}}^{700}$ enhanced the stabilization of Pb (Li et al., 2017). This findings was consistent with the H_2_O_2_ leaching experiments in Figure 4d, in which the Pb leaching content from ${BC}_{\text{Pb}}^{700}$ was lower than that of ${BC}_{\text{Pb}}^{500}$ . The underlying mechanism might be the poor stability of PbCO_3_ formed in ${BC}_{\text{Pb}}^{500}$ , and thus the Pb species transferred from F4 fraction to F3 fraction. Another potential mechanism referred to the acidity and activation of the metal (loid) s induced by the O-containing functional groups (Wang et al., 2017), which was consisitent with the change in pH value of ${BC}_{\text{Pb}}^{500}$ (Supplementary Table SA5). These results suggested that high temperature could enhance the immobilization of Pb in BC_Pb_ (Figure 3b). It should be noted that this incubation study was conducted with one soil type over 45 days. Longer-term studies across diverse soil types are needed to fully assess the long-term stability and field applicability of BC_Pb_.

### 3.5 Environmental risk assessment

**FIGURE 6:**
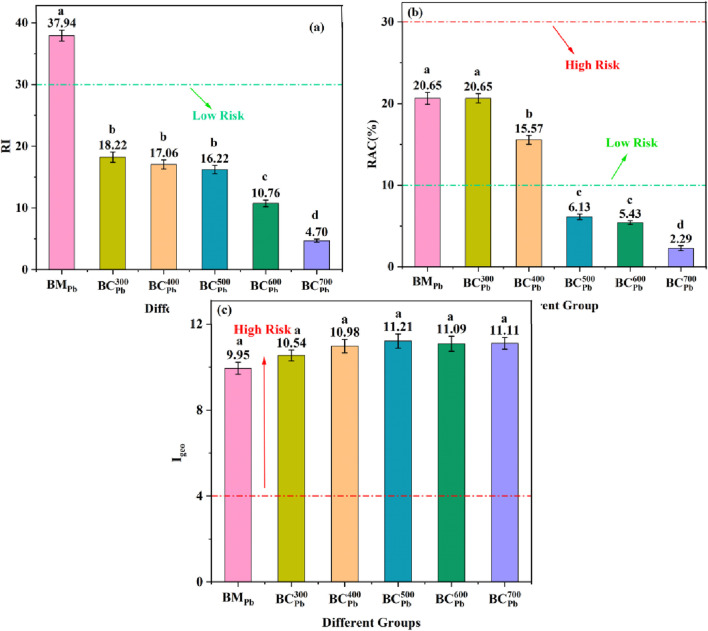
**(a)** RI, **(b)** RAC, and **(c)** I_geo_ of BM_Pb_ and ${BC}_{\text{Pb}}^{\text{CX}}$ (X = 300, 400, 500, 600, 700 °C). Error bars represent standard deviation (n = 3). Different lowercase letters above bars (or data points) indicate significant differences (p < 0.05) among groups.

As shown in Figure 6, the environmental risk indexes (RI, RAC and I_geo_) of BM_Pb_ and BC_Pb_ were calculated. The RI of BM_Pb_ and BM_H_ were 37.94 and 21.06 (Figure 6a; Supplementary Figure A9a), respectively. Additionally, the RI of BC_Pb_ and BC_H_ showed a decreasing trend with the increasing pyrolysis temperature. This suggested that the potential environmental risk of Pb gradually diminished and remained significantly below the low-risk threshold. Other studies also demonstrated that once the phytoremediation residue was pyrolyzed to biochar, its RI normally decreased to the safe threshold of ecological risk (Rodriguez-Vila et al., 2015). Similar to RI, the RAC of Pb in BM_Pb_ (Figure 6b) and BM_H_ (Supplementary Figure A9b) were 20.65% and 24.02%, respectively. The RAC values of Pb in BC_Pb_ and BC_H_ were assessed as moderate risk at 300 °C and 400 °C, whereas the values depressed to lower risk level at pyrolysis temperature ranging from 500 °C to 700 °C. Therefore, the ecological safety of Pb in BM_Pb_ could be improved by the pyrolysis method, especially at temperature exceeded 500 °C, the RAC of BC_Pb_ were much lower in comparison with the low risk standard (10%). In addition, all the I_geo_ of as prepared samples were higher than the level of severe risk (Figure 6c; Supplementary Figure A9c). Unlike the RAC and RI, which were calculated according to the chemical speciation distribution of Pb, the I_geo_ was significantly influenced by the total Pb content. Therefore, although the pyrolysis treatment could effectively depress the bioavailability of Pb, it also increased the total concentration of Pb in BC_Pb_, exhibiting obvious risk of geological accumulation. This apparent contradiction highlights a critical point: while pyrolysis successfully transformed Pb into less bioavailable forms (reducing direct toxicity as reflected by RI and RAC), the resultant high concentration of Pb in BC_Pb_ still represented a significant reservoir of the metal. This underscored the necessity for secure disposal or further treatment of BC_Pb_ to mitigate the long-term geological accumulation risk, even after pyrolysis. Overall, these results implied that pyrolysis was an efficient approach for mitigating the potential ecological hazards associated of Pb in BC_Pb_. However, the long-term geological accumulation risk of BC_Pb_ warrants continued cautious attention.

## 4 Conclusion

This study provided a systematic evaluation of pyrolysis as a stabilization treatment for Pb-enriched phytoremediation residues. The key novelty lied in elucidating the temperature-dependent mechanistic transformation of Pb speciation within biochar derived from *Iris sibirica L.*, a perennial hyperaccumulator, and establishing a direct link between the formed mineral phases (e.g., Pb_2_(P_4_O_12_), Pb_3_(CO_3_)_2_(OH)_2_) and the resulting environmental stability. The transformation of Pb from bioavailable fractions (F1+F2) to stable fractions (F3+F4) was quantitatively confirmed, with the stable portion increasing from 28.02% in raw biomass to 82.82% in ${BC}_{\text{Pb}}^{700}$ . Leaching tests and a 45-day soil incubation demonstrated that pyrolysis, especially above 500 °C, significantly reduced the direct bioavailability and short-term leaching risk of Pb, thereby offering a viable pathway to mitigate the secondary pollution hazard inherent in the storage of untreated biomass.

However, this evaluation also revealed important limitations and future challenges. The present findings, derived from a single soil type under controlled short-term conditions, required validation through long-term field studies across diverse pedological and climatic settings to confirm long-term stability. Furthermore, while the ecological risk indices (RI and RAC) were substantially lowered, the high total Pb content leading to elevated geological accumulation index (I_geo_) values indicated that the resulting biochar itself must be considered a potential source of long-term contamination and required controlled disposal or further treatment. In conclusion, pyrolysis was proven to be an effective *ex-situ* treatment for converting hazardous phytoremediation biomass into a more stable form, but its application must be tempered with considerations of cost, scale-up logistics, and the imperative for long-term environmental monitoring.

## Data Availability

The datasets presented in this study can be found in online repositories. The names of the repository/repositories and accession number(s) can be found in the article/Supplementary Material.
