# Supplementary material for: Mechanistic insights into pyrolysis temperature-dependent lead (Pb) stabilization in phytoremediation residue-derived biochar
Source: Front Chem. 2025 Nov 12;13:1705662. doi: 10.3389/fchem.2025.1705662 (PMC12647063; doi:10.3389/fchem.2025.1705662)
Supplement: Supplementary file 1 [file DataSheet1.docx]

Supporting Information

**Effect of pyrolysis temperature on the stability of lead (Pb) in biochar obtained from** **phytoremediation** **residue**

Jin Liu^1,2^, Yangyang Wang^1^, Jun Pang^3^, Jingao Wang^4^, Tongtong Li^4, *^, Lei Wang^1,*^

^1^ School of Materials and Environmental Engineering, Shenzhen Polytechnic University, Shenzhen 518055, P.R. China;

^2^ Shenzhen Minghe Science and Technology Company Limited, Shenzhen 518101, P. R. China;

^3^ College of art, Shandong Agricultural University, Tai’an 271018, P. R. China;

^4^ Technical Centre for Soil, Agriculture and Rural Ecology and Environment, Ministry of Ecology and Environment, Beijing 100012, P. R. China.

^*^ Corresponding Author: litongtong@tcare-mee.cn (T. Li); wangleicraes@163.com (L. Wang).

**Text A1 Wetland emergent plants and hydroponic culture**

*Iris sibirica* L., a common emerging plant, was used in this study. Six-month-old plants with similar biomass were selected and transplanted into the greenhouse for hydroponic cultivation. Prior to hydroponics, the plants were thoroughly rinsed with tap water to remove surface debris, followed by repeated rinsing with deionized water. The plants were cultured in polyvinyl-chloride buckets (20.0 cm diameter, 30.0 cm length), with three plants per bucket and 10.0 L of hydroponic solution. The plants were exposed to three different Pb concentrations (0.0, 0.3, 0.5 g L^-1^). For the accuracy of the experiment, plants were tested in triplicate at each Pb concentration. Deionized water was added daily to maintain a constant solution volume, and the nutrient solutions were completely replaced every week. The experiment was carried out in a greenhouse located in the Shunyi district, Beijing, lasting for 60.0 days before harvesting (from 1st June 2017 to 31st July 2017). The temperature in the greenhouse ranged from 18.0 to 22.0 °C, with an average photoperiod of 11.0/13.0 h (light/dark). The nutrient solution for culturing plants included 5.0 mmol KNO_3_, 5.0 mmol Ca(NO_3_)_2_·4H_2_O, 9.0 mmol MnCl_2_·4H_2_O, 1.0 mmol KH_2_PO_4_, 2.0 mmol MgSO_4_·7H_2_O, 0.8 mmol ZnSO_4_·7H_2_O, 0.3 mmol CuSO_4_·5H_2_O, 4.0 mmol H_3_BO_3_, 0.01 mmol H_2_MoO_4_·H_2_O and 0.8 mmol Fe_2_(SO_4_)_3_ citrate as the iron source.

**Text A2 Sample collection**

After 60.0 days of hydroponic culture, plant samples were collected from the cylinder, washed with deionized water and 0.01 M HCl, and then dissected into roots and shoots in the laboratory. Each organ was oven-dried at 70.0 °C for 72.0 h to remove moisture, ground to a fine powder using an analytical mill, and homogenized to ensure uniform element distribution. After passing through a 160.0 μm diameter sieve, the powder was weighed and prepared for subsequent Pb analysis.

## Text A3. Preparation of biochar

*Iris sibirica* L. was collected from the outskirts farm of Daxing, Beijing, China. The roots were air-dried under natural conditions, crushed by a pulverizer, and sieved through 2.0 mm mesh. The biomass was pyrolyzed into biochar using a tube furnace under nitrogen to maintain anoxic conditions. The furnace was heated to the target temperatures (300.0, 400.0, 500.0, 600.0, and 700.0 ^o^C) at a heating rate of 10.0 ^o^C·min^-1^ for 2.0 h. Biochar prepared from the root of *Iris sibirica* L. without heavy metals at different temperatures were labeled as BC300 CK, BC400 CK, BC500 CK, BC600 CK, BC700 CK respectively. Biochar prepared from *Iris sibirica* L. with Pb^2+^ stress concentration of 0.3 and 0.5 g L^-1^ were labeled as BC_Pb_ and BC_H_. In addition, BC_Pb_ and BC_H_ derived at differ temperatures were labeled as BCX Pb and BCH Pb, respectively, wherein X represent pyrolysis temperature (300.0, 400.0, 500.0, 600.0, and 700.0 ^o^C).

**Text A4. Pb content and Ref**

The root of *Iris sibirica* L. and the derived biochar samples were digested in a concentrated acid mixture (HNO_3_: HCl = 3: 1, v/v) using a laboratory microwave digestion system (Milestone, Co., Italy) equipped with an internal temperature sensor. A mixture containing 0.2 g of dried sample and 12.0 mL of concentrated acid was added to a 100.0 mL closed Teflon digestion tank. The digestion temperature was set to 180.0 °C for 12.0 h. After cooling to room temperature, the solution was diluted to 100.0 mL with 1% HNO_3_ for content analysis. Quality assurance and quality control (QA/QC) was performed using the certified reference material GBW07407 (soil). The measured recovery of Pb was between 95% and 105%, confirming the accuracy of the total digestion method. All samples were analyzed in triplicate, and the relative standard deviation was below 5%. Next, a high performance inductively coupled plasma-mass spectrometer (ICP-MS) (Ne_x_ION 300, PE Company, USA) was used to analyze the total content of the heavy metals. Each experiment was repeated three times. The relative enrichment coefficient (Ref) of heavy metals in biochar was calculated using the following formula.

| $Ref=\frac{C_{2}}{C_{1}}$ | (1-1) |
| --- | --- |

Here, C_1_ and C_2_ are the concentrations of heavy metals in biomass and biochar respectively.

**Text A5. Heavy metal** **speciation by BCR sequential extraction**

The fractions of Pb speciation in the biomass of *Iris sibirica* L*.* and its pyrolysed biochars were measured using the BCR sequential extraction method (Cuong and Obbard, 2006). The specific extraction steps were as follows[1-3]:

Step 1 (F1): 0.5 g of dried sample and 20.0 mL of acetic acid (0.1 mol·L^-1^) were added to a 100.0 mL polypropylene centrifuge tube and shaked at 180 rpm, 25.0 °C for 16.0 h. The mixture was centrifuged at 4000 rpm for 20.0 min, and the supernatant was filtered through a 0.45μm nylon membrane.

Step 2 (F2): 20.0 mL of hydroxylamine hydrochloride (NH_2_OH·HCl, 0.1 mol·L^-1^, pH = 2.0) was added to the residue from Step 1 and subjected to the same shaking, centrifugation, and filtering conditions to obtain the reducible oxides.

Step 3 (F3): 10.0 mL H_2_O_2_ (30%, pH = 2.2) was added to the residue from the Step 2 with intermittent manual stirring at 25.0 °C for 1.0 h, then the mixture solution were placed in a water bath at 85.0 °C for another 1.0 h. When the solution volume was reduced to below 1.5 mL, 5.0 mL of H_2_O_2_ was added again and heated to near dryness. After cooling to room temperature, 50.0 mL of ammonium acetate (NH_4_Ac, 1.0 mol·L^−1^, pH = 2.0) was added, and the mixture was stirred at 25 °C for 16.0 h. The solution was centrifuged to obtain the oxidizable fraction bound to organic matter.

Step 4 (F4): The residue from Step 3 was dissolved in acid solution (HNO_3_:HCl = 3:1, v/v) to obtain the residue fraction.

The recovery rates for the entire BCR sequential extraction procedure were calculated and found to be in the acceptable range of 90% to 102%. The consistency of these recoveries supports the validity of the speciation data obtained.

## Text A6. Simulated ageing of biochar

The leaching behavior of Pb in the biomass of *Iris sibirica* L*.* and its pyrolysed biochars was investigated under different environmental conditions. Four types of ageing conditions were simulated in the laboratory, including deionized water extraction (pH = 7.03, HJ557-2009, China), acidification leaching, oxidation leaching and the toxicity characteristic leaching procedure (TCLP) (pH = 2.88 ± 0.05, US EPA Method 1311), which were commonly used to evaluate the leaching toxicity of solid waste.

**Deionized water leaching procedure:** 0.2 g biomass or its pyrolysed biochars was placed into a 250.0 mL conical flask containing 100.0 mL deionised water (pH = 7), sealed and shaked for 8.0 h in an incubator (200 r·min^-1^, 25.0 ^o^C). The supernatant was then extracted and filtered through a 0.45 μm membrane before ICP-MS analysis.

**Acid leaching procedure:** Based on the deionized water leaching procedure, the 100.0 mL of deionized water was replaced with solutions of pH 2.0, 4.0, 6.0, 8.0, and 10.0 to simulate the effects of long-term exposure to acid conditions.

**Oxidation leaching procedure:** Based on the deionized water leaching procedure, the 100.0 mL deionised water was replaced with 3%, 5%, 10% and 20% H_2_O_2_ solutions.

**Toxicity characteristic leaching procedure:** 1.0 g biomass or its pyrolysed biochars was mixed with 20.0 mL of extraction solution (acetic acid, pH = 2.88±0.05) and shaken for 18.0 h (30 r·min^-1^, 25.0 ^o^C). The supernatant was extracted and filtered through a 0.45 μm membrane before ICP-MS analysis.

Each leaching experiment was repeated three times. The Pb leaching rate was calculated by the following formula:

|  | $R=\frac{w\times c_{1}}{w\times c_{2}}\times100\%$ | (1-3) |
| --- | --- | --- |

Here, R is the leaching rate of Pb (%). C_1_ is the leaching concentration of Pb (mg·kg^-1^). C_2_ is the total content of Pb (mg·kg^-1^). W is the mass of biomss or biochar (g).

## Text A7. Soil pretreated process

Soil was collected from Daxing District, Beijing, China. Firstly, the soil was sieved through a 100-mesh screen and sterilized by UV light for 10.0 h to eliminate the microbial influences. Secondly, the treated soil was placed in polyvinyl-chloride buckets (20.0 cm diameter, 30.0 cm length) and sprayed with water every two days to maintain the 60% soil humidity during the experiments.

Basic physicochemical properties of soils: pH = 6.39, organic matter = 12.03 mg·g^-1^, TN = 0.52 mg·g^-1^, TP = 3.34 mg·g^-1^.

**Text A8. Assessment of potential environmental risks of heavy metals in biochar**

The potential ecological risk assessment index (*RI*), Muller geological accumulation index (*I_geo_*) and risk assessment code (*RAC*) were used to assess the potential ecological risk of heavy metals according to **Eq. (1-3)**, **Eq. (1-4)** and **Eq. (1-5)** [4, 5].

*RI* = ∑E_r_ = ∑T_r_ × C_f_ = ∑T_r_ × C_i_ / C_n_ (1-3)

*I_geo_* = log_2_ (C_n_ / 1.5B_n_) (1-4)

*RAC* = F_1_ / C_n_ × 100% (1-5)

Wherein C_f_ is the heavy metal contaminated factor, C_i_, C_m_ and C_n_ are the potential migrating species (F1, F2 and F3), stable species (F4) and total Pb, respectively, B_n_ is the background value of Pb in the soil (24.60 mg·kg^-1^), T_r_ is the toxicity factor for Pb (5.00).

***RAC* Risk Levels:** *RAC* ≤ 1% represents no risk, 1% < *RAC* ≤ 10% represents low risk, 11% < *RAC* ≤ 30% represents medium risk, 31% < *RAC* ≤ 50% represents high risk, and *RAC* > 50% represents severe risk.

***RI* Risk Levels:** *RI* < 30 represents no risk, 30 ≤ *RI* < 60 represents medium risk, 60 ≤ *RI* < 120 represents relatively high risk, and *RI* > 120 represents very high risk.

***I_geo_* Risk Levels:** *I_geo_* ≤ 0 represents no risk, 0 < *I_geo_* ≤ 1 represents no risk to medium risk, 1 < *I_geo_* ≤ 2 represents medium risk, 2 < *I_geo_* ≤ 3 represents medium risk to high risk, 3 < *I_geo_* ≤ 4 represents high risk, 4 < *I_geo_* ≤ 5 represents high to severe risk, and *I_geo_* > 5 represents severe risk.

## Text A9. Semi-quantitative analysis of Pb species based on XRD peak areas

A semi-quantitative analysis of the crystalline Pb species identified in the XRD patterns (Figure 1b) was performed by measuring the integrated area of their primary diffraction peaks using Jade software. The relative abundance of each phase was calculated based on the proportion of its peak area to the total area of all identified Pb-related peaks. It is important to note that this method provides an approximation, as differences in scattering factors among different crystalline phases are not accounted for. The results, presented in Table A3, illustrate the temperature-dependent transformation of Pb speciation.


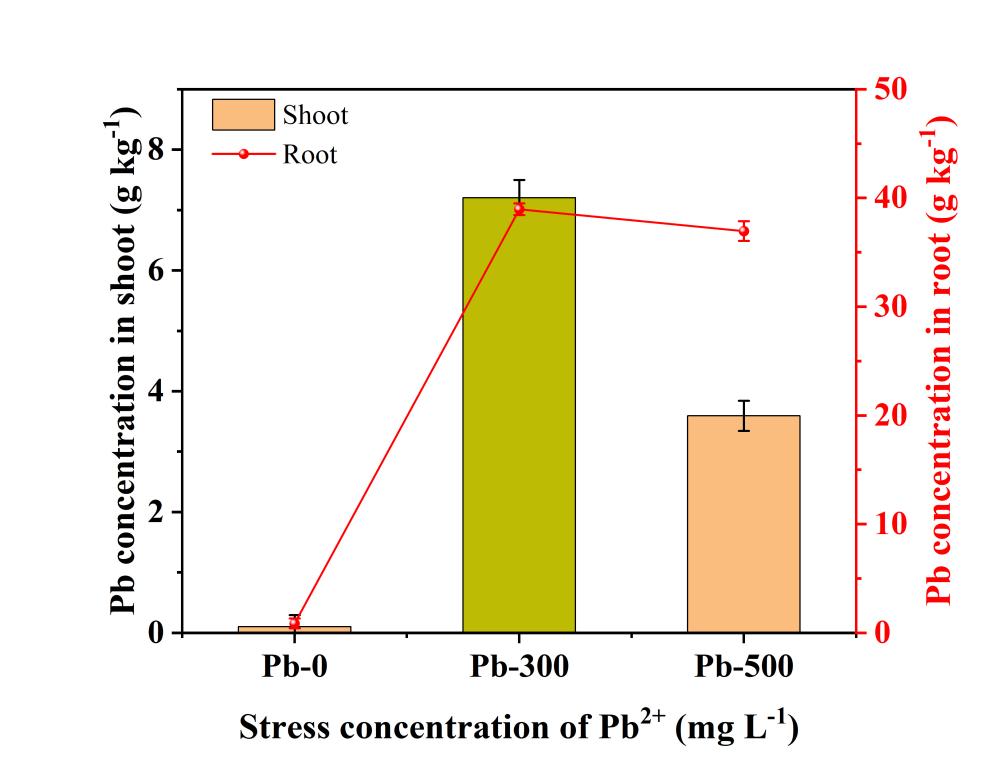


**Figure A1. Concentration of Pb ions in biomass**


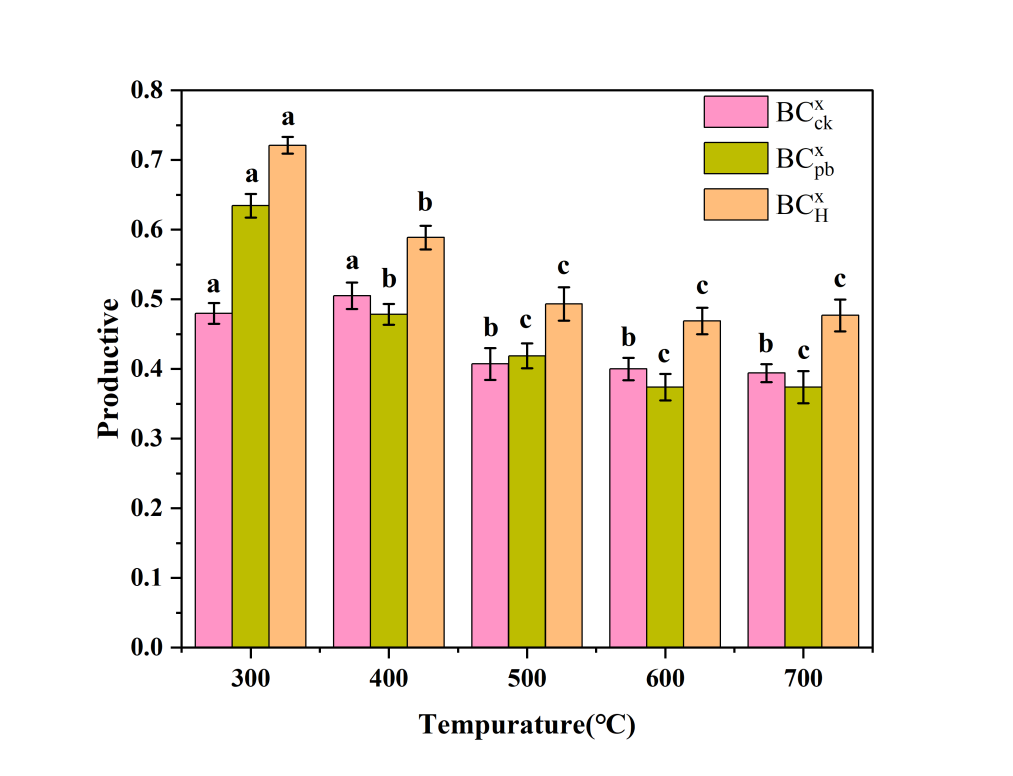


**Figure A2. Yield of biochar produced at different pyrolysis temperatures. Error bars represent standard deviation (n=3). Different lowercase letters above bars (or data points) indicate significant differences (p < 0.05) among groups.**


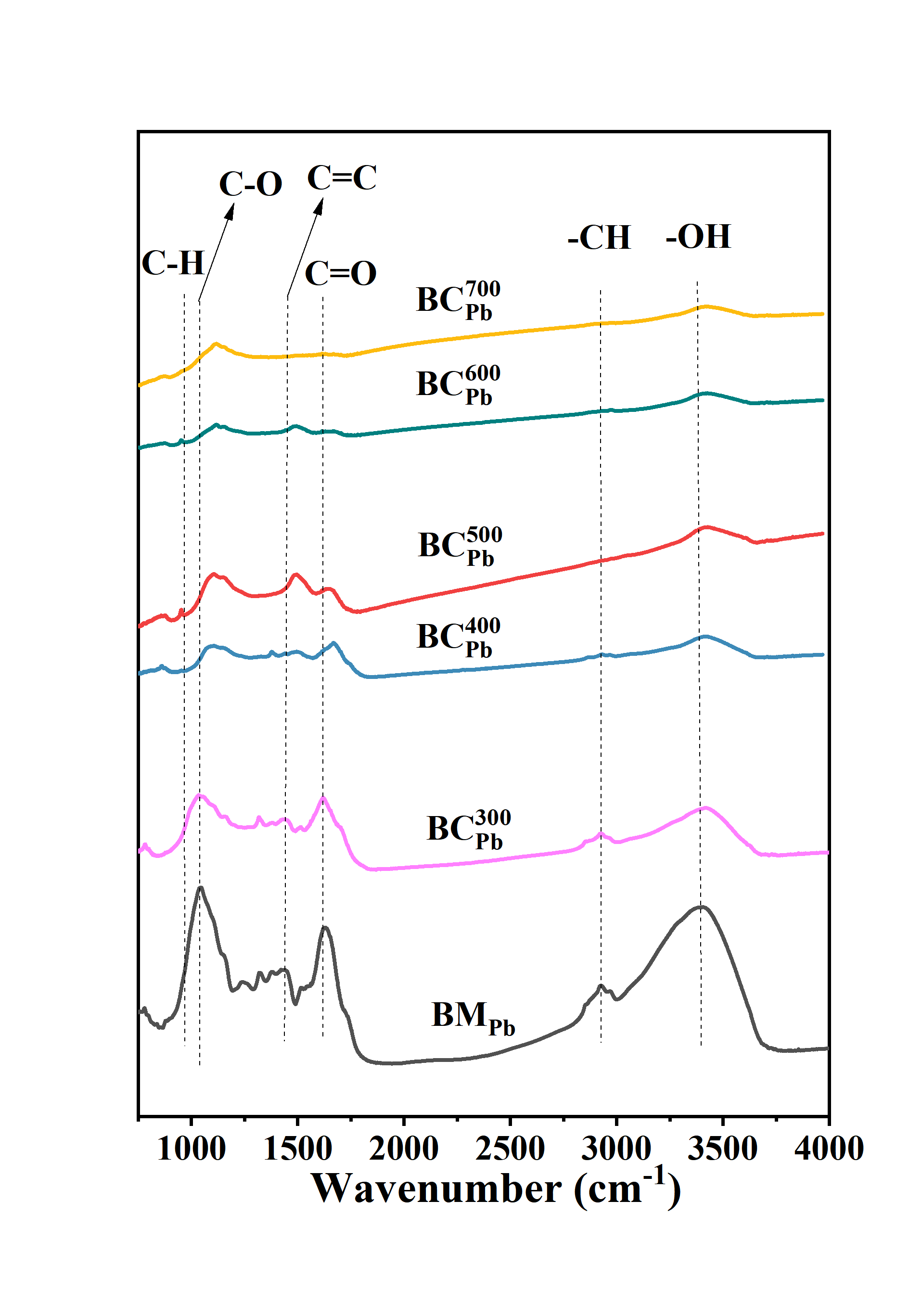


**Figure A3. FTIR spectra of BM_H_ and its biochar**


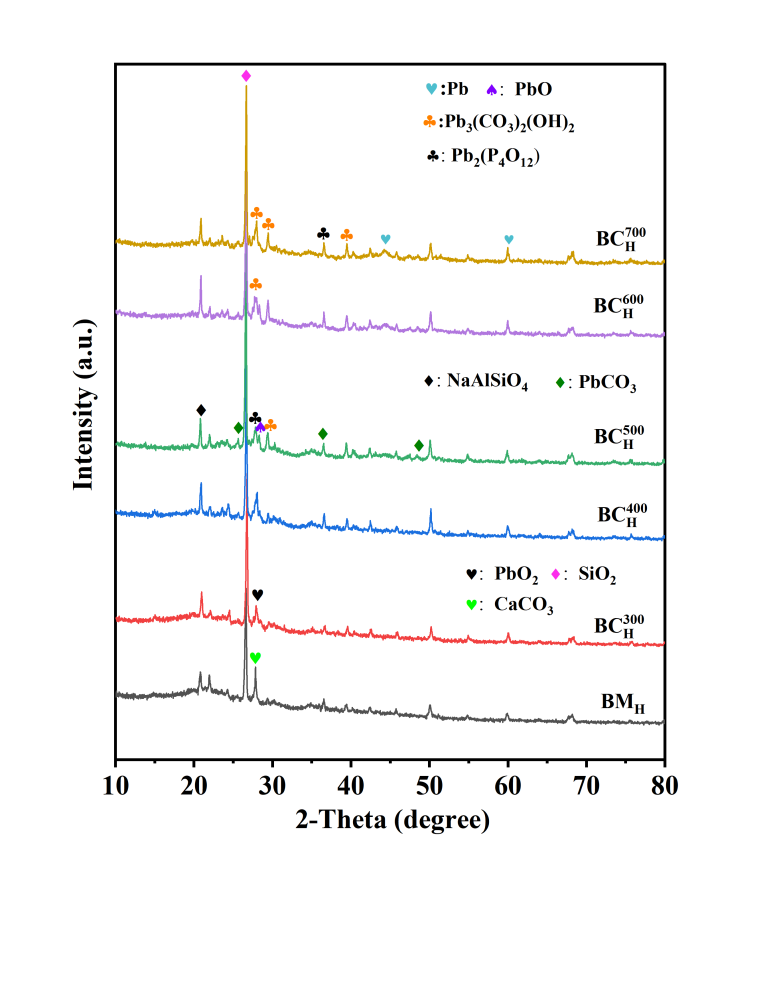


**Figure A4. X-ray diffraction pattern of BM_H_ and its biochar**


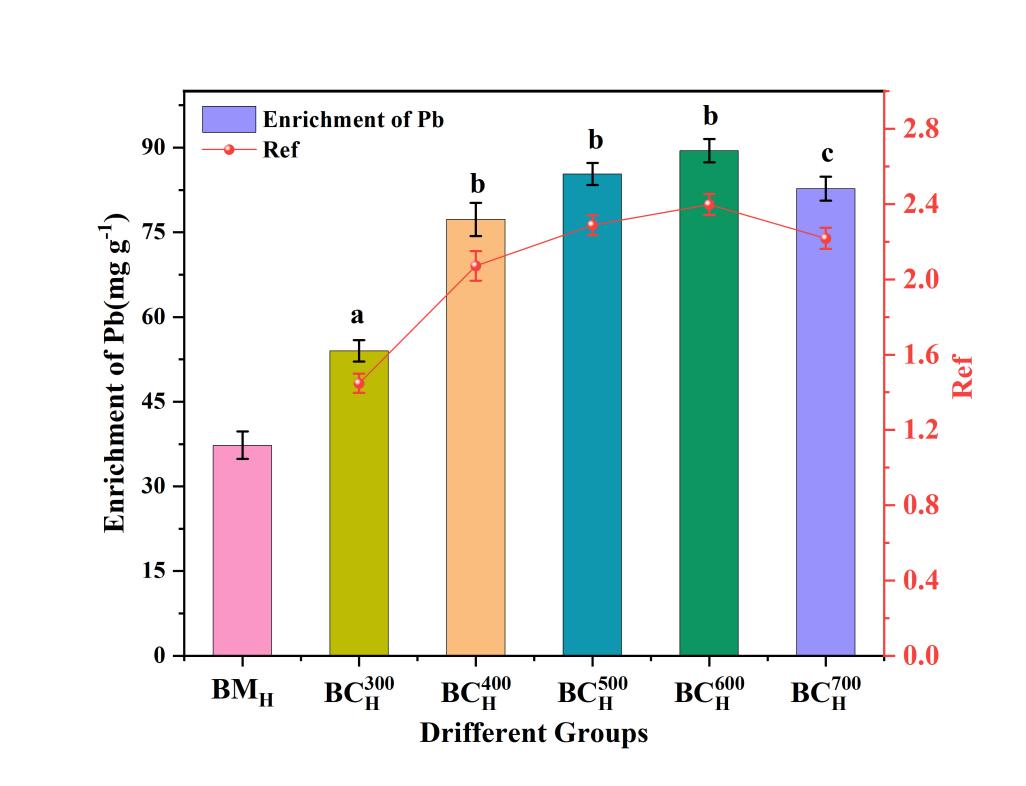


**Figure A5. The content of Pb and relative enrichment factors in BM_H_ and its biochars. Error bars represent standard deviation (n=3). Different lowercase letters above bars (or data points) indicate significant differences (p < 0.05) among groups.**


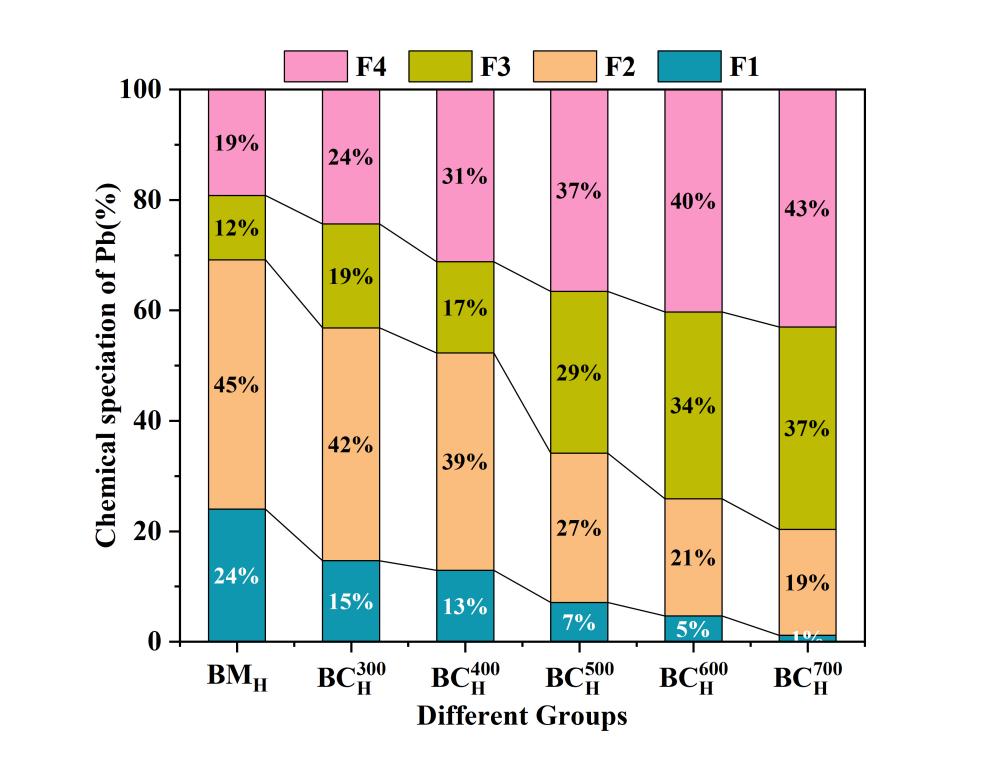


**Figure A6. Chemical speciation of Pb in BM_H_ and BCX H.**


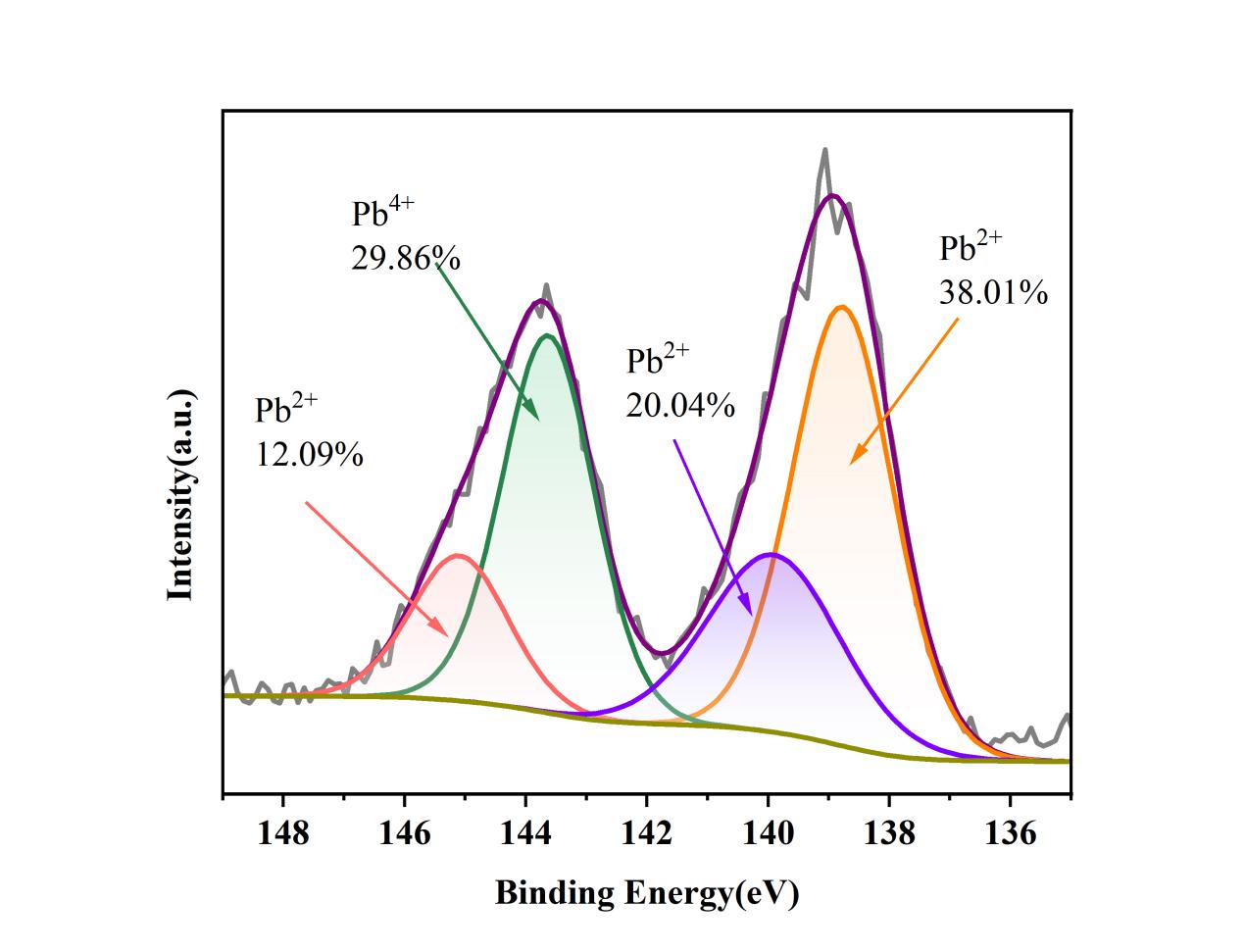


**Figure A7. Pb 4f XPS spectra of BC500 Pb post-BCR residue**


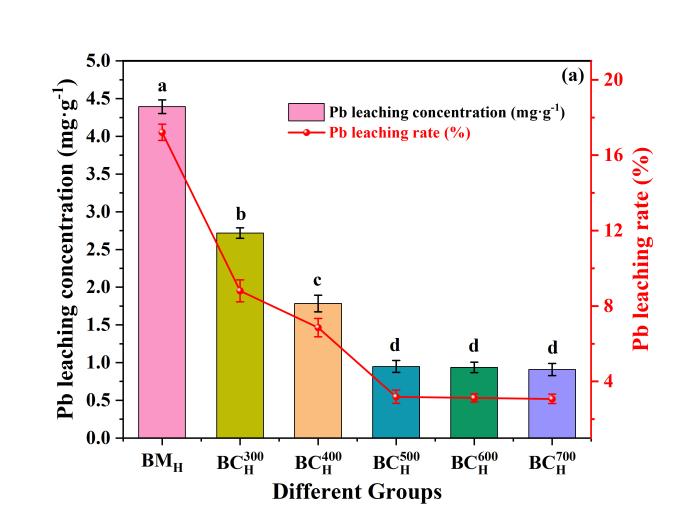

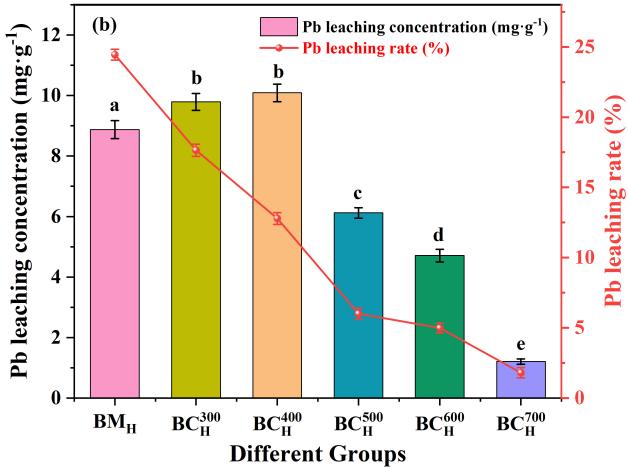


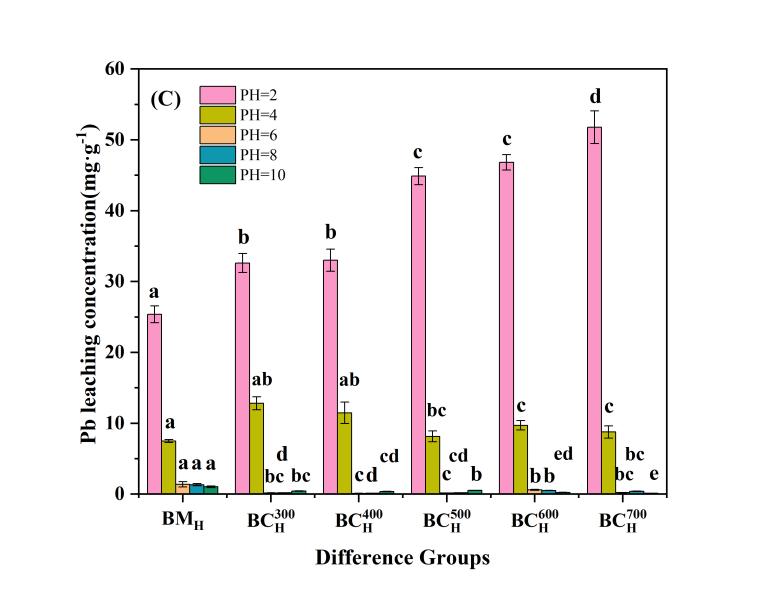

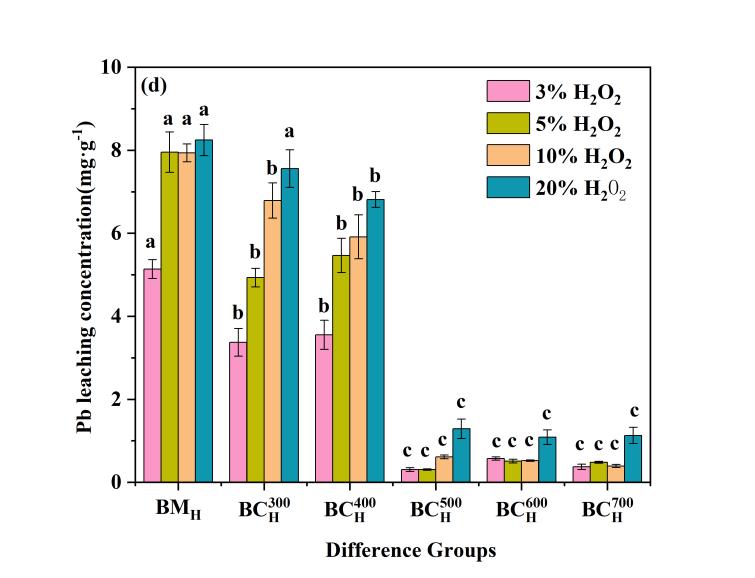


**Figure A8. Pb leaching concentration of BM_H_ and BCX H in (a) deionized water, (b) TCLP, (c) pH and (d) H_2_O_2_ conditions. Error bars represent standard deviation (n=3). Different lowercase letters above bars (or data points) indicated significant differences (p < 0.05) among groups. For Figure (c) and (d), the statistical comparisons and letter designations were independent for each pH and H_2_O_2_ condition.**


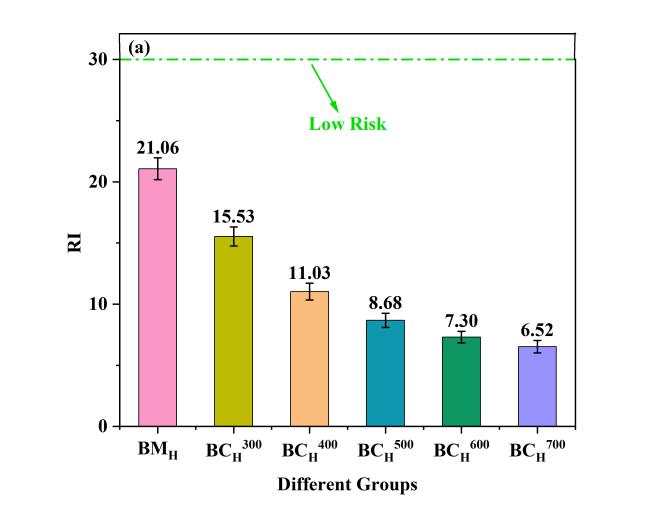

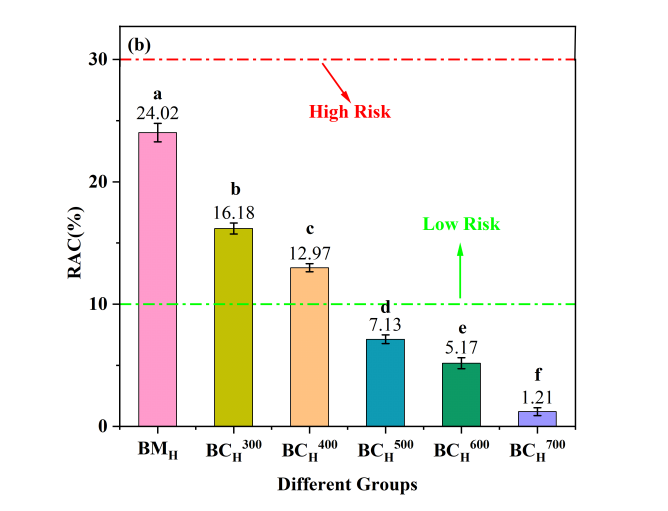

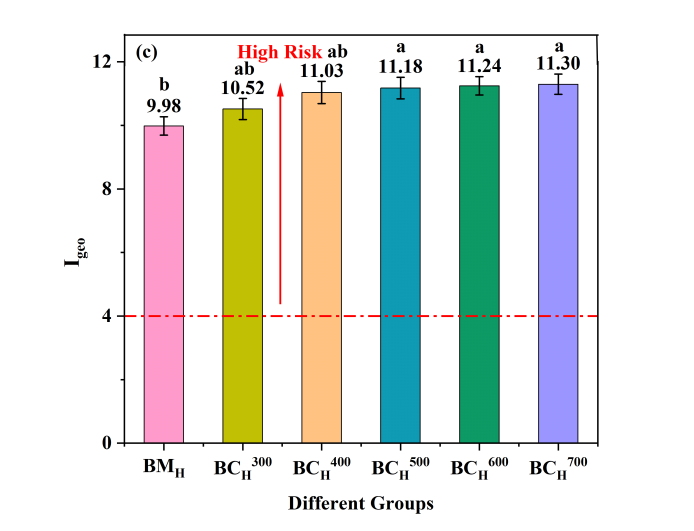


**Figure A9. (a) *RI*, (b) *RAC*, and (c) *I_geo_* of BM_H_ and BCX H. Error bars represent standard deviation (n=3). Different lowercase letters above bars (or data points) indicate significant differences (p < 0.05) among groups.**

**Table A1 Ash content, yield rate and pH of biochar.**

| **Pyrolysis temperature**  **(°C)** | **Ash content**  **(%)** | **Yield rate**  **(%)** | **pH** |
| --- | --- | --- | --- |
| **BM_CK_** | - | - | 7.25 ± 0.24 |
| **BC** | 14.53 ± 0.31 | 47.98 ± 0.26 | 7.63 ± 0.28 |
| **BC** | 25.47 ± 0.23 | 50.49 ± 0.11 | 9.02 ± 0.25 |
| **BC** | 31.34 ± 0.19 | 40.72 ± 0.39 | 9.34 ± 0.16 |
| **BC** | 39.54 ± 0.22 | 39.99 ± 0.16 | 9.54 ± 0.17 |
| **BC** | 44.27 ± 0.18 | 39.40 ± 0.27 | 9.84 ± 0.22 |
| **BM_Pb_** | - | - | 6.93 ± 0.18 |
| **BC** | 26.93 ± 0.41 | 63.44 ± 0.23 | 8.40 ± 0.13 |
| **BC** | 34.33 ± 0.21 | 47.82 ± 0.41 | 9.62 ± 0.28 |
| **BC** | 43.85 ± 0.33 | 41.88 ± 0.44 | 9.59 ± 0.21 |
| **BC** | 47.77 ± 0.35 | 37.40 ± 0.13 | 9.93 ± 0.35 |
| **BC** | 54.72 ± 0.39 | 37.39 ± 0.21 | 10.49 ± 0.32 |

**Table A2. The correlation between ash content and pH was calculated by the Spearman model.**

|  | **Ash content**  (correlation coefficient/P) | **pH**  （correlation coefficient/P） |
| --- | --- | --- |
| **Ash content** | 1 (0.000***) | 0.893 (0.019***) |
| **pH** | 0.893 (0.019***) | 1 (0.000***) |

Note：***, **, * represents significance levels of 1%, 5%, and 10%, respectively

Spearman's rank correlation coefficient is a nonparametric statistical measure used to evaluate the dependence of two variables. Spearman's correlation coefficient (*r_s_*) ranges from -1 to 1, where *r_s_* > 0 indicates a positive correlation and *r_s_* < 0 signifies a negative correlation. The magnitude of *r_s_* (|*r_s_*|) determines the strength of the correlation between the variables. This method is particularly advantageous when the data do not meet the assumptions required for parametric tests, such as normality or linearity, making it a robust tool for analyzing non-linear or ordinal data relationships.

The results above showed that ash content was significantly correlated with pH value.

**Table A3. Semi-quantitative distribution (%) of crystalline Pb species in BC_Pb_ at different pyrolysis temperatures.**

| **Pyrolysis Temperature (**°C**)** | **PbO_2_** | | **PbO** | **Pb_2_(P_4_O_12_)** | **PbCO_3_** | **Pb_3_(CO_3_)_2_(OH)_2_** | **Pb⁰** |
| --- | --- | --- | --- | --- | --- | --- | --- |
| 300 | | 100.00 | 0.00 | 0.00 | 0.00 | 0.00 | 0.00 |
| 400 | | 0.00 | 54.85 | 12.13 | 33.02 | 0.00 | 0.00 |
| 500 | | 7.47 | 8.00 | 43.73 | 21.78 | 19.02 | 0.00 |
| 600 | | 0.00 | 10.08 | 45.24 | 14.68 | 30.00 | 0.00 |
| 700 | | 0.00 | 0.00 | 32.82 | 0.00 | 29.87 | 37.31 |

**Table A4. Final pH of Leaching Solution after 8-hour Incubation with BC_Pb_**

| **Initial Leaching Solution pH** | **Biochar Sample** | **Final pH of Supernatant** |
| --- | --- | --- |
| 2.0 | BC300 Pb | 3.2 ± 0.3 |
| 2.0 | BC700 Pb | 5.8 ± 0.4 |
| 4.0 | BC300 Pb | 5.5 ± 0.2 |
| 4.0 | BC700 Pb | 7.1 ± 0.3 |
| 6.0 | BC700 Pb | 8.5 ± 0.2 |

**Table A5. Changes of pH in soil after the addition of BM_Pb_ and BCX Pb**

| Exogenous Additives | Cultivation time (d) | | | | | |
| --- | --- | --- | --- | --- | --- | --- |
|  | 0 | 3 | 7 | 15 | 30 | 45 |
| Blank | 6.39 | 6.37 | 6.38 | 6.38 | 6.38 | 6.39 |
| BM_Pb_ | 6.23 | 6.64 | 6.65 | 6.67 | 6.68 | 6.68 |
| BC300 Pb | 6.61 | 6.69 | 6.73 | 6.69 | 6.69 | 6.73 |
| BC500 Pb | 6.72 | 6.74 | 6.77 | 6.71 | 6.69 | 6.75 |
| BC700 Pb | 6.78 | 6.72 | 6.76 | 6.76 | 6.82 | 6.86 |

**References**

1. He, E., et al., Two years of aging influences the distribution and lability of metal (loid) s in a contaminated soil amended with different biochars. Science of the Total Environment, 2019. 673: p. 245-253.
2. Zhang, W., et al., Rice waste biochars produced at different pyrolysis temperatures for arsenic and cadmium abatement and detoxification in sediment. Chemosphere, 2020. 250: p. 126268.
3. He, E., et al., Two years of aging influences the distribution and lability of metal (loid) s in a contaminated soil amended with different biochars. Science of the Total Environment, 2019. 673: p. 245-253.
4. Zhao, S.; Feng, C.; Yang, Y., et al., **2012**. Risk assessment of sedimentary metals in the Yangtze Estuary: new evidence of the relationships between two typical index methods. J. Hazard. Mater. 241-242, 164-72.
5. Zhang, Y.; Chen, Z.; Xu, W., et al., **2020**. Pyrolysis of various phytoremediation residues for biochars: Chemical forms and environmental risk of Cd in biochar. Bioresour Technol. 299, 122581.
